# What motivates doctors to leave the UK NHS for a “life in the sun” in New Zealand; and, once there, why don’t they stay?

**DOI:** 10.1186/s12960-015-0069-4

**Published:** 2015-09-08

**Authors:** Robin Gauld, Simon Horsburgh

**Affiliations:** Centre for Health Systems, Department of Preventive and Social Medicine, University of Otago, PO Box 56, Dunedin, 9010 New Zealand

**Keywords:** Medical migration, UK NHS, New Zealand, Survey

## Abstract

**Background:**

At 44%, New Zealand has the highest proportion of international medical graduates (IMGs) in its workforce amongst OECD member countries. Around half of New Zealand’s IMGs come from the UK NHS, yet only around 50% stay longer than 1 year post-registration with significant costs to the New Zealand health care system. Why these doctors go to New Zealand and do not stay for long is an important question.

**Methods:**

UK-trained doctors who had gained registration with the Medical Council of New Zealand and currently practising in New Zealand were surveyed (*n* = 1357) on the motivation for their move to New Zealand, experiences once there and what was prompting any intentions to move away from New Zealand. Multivariate proportional odds models (POM) were used to quantify various associations.

**Results:**

The survey had a 47% response (*n* = 632). Quality of life considerations motivated 96% of respondents to move to New Zealand, although 65% indicated they were pushed by a desire to leave the NHS. POM analyses revealed older respondents were significantly less likely than younger respondents to be motivated by quality of life considerations. Younger doctors were significantly more likely to be seeking to leave the NHS. Seventy-six per cent of respondents signalling an intention to leave New Zealand indicated that the desire to return to the UK was the primary reason for this.

**Conclusion:**

There is a long history of medical migration from the UK to New Zealand. However, the 65% of respondents in this study seeking to leave the NHS was much higher than found elsewhere, perhaps reflecting increasing workplace and funding pressures in recent years. Of concern to policy makers were the higher odds of seeking to leave the NHS motivating younger doctors. Various changes “down under”, in New Zealand as well as Australia, mean their IMG markets may well be tightening up.

## Background

The challenge of maintaining a stable and balanced medical workforce is common to most countries. It is particularly so for low- and middle-income countries where working conditions, income, training opportunities and lifestyle are routinely cited as reasons for why it is difficult to retain a full and sustainable workforce in the face of the considerable global opportunities available to doctors [1]. A 2014 review reiterated that such challenges also confront the high-income countries of the OECD with only 1 of 34 member countries (the Netherlands) suggesting that they had no particular issue with doctor supply or distribution [2]. A majority of these countries face a range of medical workforce challenges with questions over how they will meet increasing public demand for health care, maintain a sufficient number of general practitioners (GPs), fill shortages in particular hospital specialties and, very importantly, ensure an even distribution of doctors across the population. One response is to seek the services of international medical graduates (IMGs), doctors born or trained abroad who have specific desired skills or may be willing to work in hard-to-staff areas [3].

Data show that IMGs have long played an important role in many countries; in several, this role has grown in recent years [2-4]. The key reason for this is the basic requirement for more doctors to keep up with ever-increasing health care demand which is not able to be met due to shortfalls in the numbers of locally trained doctors being produced. IMGs are also often in demand to take the place of locally trained doctors who themselves join the ranks of IMGs [5]. The global movement of doctors has various benefits, particularly in terms of training opportunities that may not be available in the IMG’s country of origin, and provides all-important experience working within different health systems and populations.

A range of studies have investigated factors that drive doctor migration [6-10]. These studies have largely focused on the flow of doctors from low to higher income destinations. They have identified a series of factors that “push” doctors away from their country of origin, including substandard working conditions and facilities, inadequate pay, high-stress levels, lack of clinical and administrative support and a poor quality or unsafe living environment. Coupled with this are factors that “pull” doctors into a new country, such as quality of life, promise of better working conditions, higher incomes often driven by staff shortages in destination countries and training opportunities.

Researchers have also investigated the broader implications of migration. IMGs are often entering a health system and country quite different from their origin. This can lead to challenges of “cross-cultural adaptation”, pointing to a need for additional support to help IMGs integrate into their new role and society [11]. Allegations of discrimination against IMGs, especially those from ethnic minority groups, in the medical registration process and workplace are not uncommon [12]. An Australian study of patient complaints and adverse disciplinary findings found that there was a significantly higher incidence amongst IMGs than locally trained doctors but also that such risks differed markedly by the doctors’ country of origin [13]. In many parts of the world, the pull of particular destination countries can be a major contributor to workforce shortages in feeder countries, raising various ethical and policy questions emphasized by researchers and addressed in the World Health Organization’s 2010 *Global Code of Practice on the International Recruitment of Health Personnel* [10,14-17].

There has been a gradual increase in research into doctor migration between higher income countries, with Zubaran providing a useful historical summary [18]. More recently, a WHO project resulted in a series of book chapters published in 2011 investigating the patterns of migration and mobility of health professionals, including doctors, within the EU area and the impacts of this [19]. In 2014, the OECD published a working paper describing the “geographic imbalances” in doctor supply in which various policy responses were outlined [2]. An earlier study looked at why UK-trained doctors migrate to New Zealand, drawing on a survey undertaken in 2009 [20]. This article seeks to contribute to this body of research. It probes the motives of UK-trained doctors who have migrated to New Zealand, their experiences in New Zealand and reasons for departing again. In doing so, it presents findings from a 2014 survey of UK-born or UK-trained doctors working in New Zealand.

The UK–New Zealand case is important for several reasons. First, the proportion of IMGs in New Zealand’s doctor workforce, at 44% in 2014, is the highest of any OECD country. A 2008 OECD report emphasized New Zealand’s heavy reliance on migrant health professionals and described a series of related concerns [21]. Despite this report, and its recommendations for improving the situation, the proportion of IMGs in New Zealand’s workforce has continued to grow [22]. The 2008 report also noted that New Zealand is amongst the highest per capita doctor-exporting countries in the OECD, with local graduates routinely going abroad in search of new opportunities, especially to Australia. The report similarly made note of onward migration, of IMGs coming initially to New Zealand then moving to Australia [21]. Second, while New Zealand has long relied on IMGs, its health system would not function without them meaning there is a structural shortage of locally trained doctors [22]. Third, around half of New Zealand’s 3500 current IMGs hail from the UK, and they go into a health system not dissimilar to the UK NHS (Box 1) [23]. A 2014 UK General Medical Council report noted that some 61% of its certificates of good standing were issued to doctors with addresses in Australia or New Zealand [24], leading one newspaper to suggest that “They cost GBP610,000 to train, but 3,000 a year are leaving us for a life in the sun…” [25]. Of these, roughly 500 per annum seek work in New Zealand [26]. Coupled with this are reports of an increasingly “stressed, exhausted, burnt out” NHS staff [27]. Fourth, recruiting NHS doctors is not a sustainable solution to New Zealand’s workforce shortages. A year after registration, only 53% of UK doctors remain in New Zealand, dropping to 30% after 2 years and 20% after 8. By contrast, 70% of New-Zealand-trained doctors are still there after 8 years, suggesting that a locally grown workforce is more likely to contribute to sustainability [23,26]. The costs of medical migration for New Zealand, and countries facing similar challenges, are high. These include recruitment and associated costs such as relocation, locum coverage for vacant posts and a period of supervision by a registered and practising doctor of new IMGs seeking medical registration [22,31].

**Table:** 

**Box 1 The New Zealand health system** New Zealand’s 1938 Social Security Act was the world’s first attempt to create a “national health service”. Doctor resistance meant the intent was never realized. Public hospitals salary all staff and are free of patient charges. General practitioners are largely in private practice and act as gatekeepers. They receive considerable government subsidies but charge most patients a fee per consultation, creating an access barrier [28]. The Government contributes 83% of total health expenditure, as in the UK. Around 40% of public hospital specialists have a separate private practice, often considered to be attractive in terms of public sector workforce retention [29]. The parallel private system means patients of better means are able to circumvent public hospital waiting times or access treatments considered to be lower priority in the constrained public sector [30].	

## Methods

In mid-2014, all UK-born or UK-trained IMGs who had registered with the Medical Council of New Zealand within the previous 10 years, and were currently practising and with an active email address, were invited to complete an online survey (*n* = 1357). The survey included themed sections, each containing a series of fixed-response Likert-type scale items in a particular area of inquiry as described below. The survey drew on and adapted items developed for similar workforce migration surveys which had used fixed-response scales [32-35]. Questions were also developed specifically to address issues known to be of concern to the Medical Council of New Zealand, which is responsible for registration and oversight of professional standards for IMGs, and to New Zealand policy makers who were interviewed prior to survey tool development. These questions mostly focused on motivation to leave New Zealand. The first section of the survey inquired into motivations for the move to New Zealand. The second section addressed factors associated with working in New Zealand. A third section was dedicated specifically to respondents who indicated that they were considering moving away from New Zealand, inquiring into motivations for this. A final section, not reported on in this article, probed mechanisms for respondents to translate the knowledge that they bring with them into the New Zealand health system and services. The survey also contained background questions. Survey piloting was limited to a small group including clinical leaders and others at the Medical Council; a version of the survey designed for the nursing workforce was circulated amongst managers and clinical leaders of the national nurses union.

An email invitation to participate in the survey was sent by the Medical Council of New Zealand to those in the sample, with an embedded link to the survey website which was managed by the authors at the University of Otago. One reminder email was sent 2 weeks after the initial invite. Respondents were able to complete the survey anonymously. Any identifying information provided by participants was removed prior to analyses and stored separately from the participant’s data. The study protocol and survey were reviewed and approved by the University of Otago Human Research Ethics Committee.

Data were analysed using R version 3.1.2 [36]. Multivariate proportional odds models (POMs) were used to quantify the associations between the participant characteristics of gender, age, years of experience and main form of medical work (general practitioner, hospital specialist, registrar or other) and responses on the Likert scale items. The models were fitted using the R package *ordinal* (*version 2015.1-21*) with the flexible threshold option [37]. These models yielded odds ratios (ORs), with an OR greater than 1 indicating an increased likelihood of responding towards the positive end of the Likert scale compared to the reference group. An OR less than 1 indicated the converse.

## Results 

The survey had a 47% (*n* = 632) response rate, with 97% of respondents completing the survey in full. Table 1 lists key characteristics of respondents, which were similar to those of non-respondents. Although the demographics of those intending to leave and all respondents appear to be quite different, a multiple logistic regression model including all of the demographic factors listed (with years practising in New Zealand dichotomized to “Five or less years” and “Six or more years”) only found that respondents practising in New Zealand for six or more years were less likely to be considering moving away (odds ratio = 0.39, 95% confidence interval 0.23–0.65, *P* = 0.0003), while respondents in the five or less years job category were more likely to be considering moving away (odds ratio = 3.40, 95% confidence interval 1.69–6.82, *P* = 0.0006).

**Table 1:** **Demographic characteristics of the survey respondents (** ***n*** **= 632), the UK-trained doctor workforce in New Zealand (** ***n*** **= 1357) and survey respondents who had indicated that they were considering moving from New Zealand (** ***n*** **= 164)**

	**Survey respondents**		**UK-trained workforce**		**Respondents considering moving away**	
	***n***	**%**	***n***	**%**	***n***	**%**
Gender						
Female	318	50	678	50	80	49
Male	312	50	679	50	84	51
Age						
20–30	170	27	463	34	81	49
31–40	244	39	550	41	49	30
41–50	142	23	241	18	19	12
51–60	51	8	75	6	12	7
61+	21	3	28	2	3	2
Main job^a^						
General practitioner	133	21	241	18	15	9
Hospital specialist	176	28	282	21	36	22
Registrar	221	35	605	45	63	38
Other	101	16	229	17	50	30
Total years practising						
5 or less years	140	23	403	30	72	44
6–10 years	188	30	390	29	43	26
11–15 years	96	15	204	15	17	10
16–20 years	64	10	149	11	9	5
21 or more years	133	21	211	16	23	14
Total years practising in NZ						
5 or less years	410	65	–	–	140	85
6–10 years	212	34	–	–	24	15
11–15 years	3	0	–	–	0	0
16–20 years	2	0	–	–	0	0
21 or more years	2	0	–	–	0	0
Total	632	100	1357	100	164	100

Information on years practising in New Zealand was not available.

^a^In New Zealand, “specialists” are interchangeably called “consultants”. “House surgeons/officers” were not included in the survey sample as it is unusual for an IMG to hold such a post where preference is given to New Zealand graduates.

Table 2 lists responses to the items on motivations to migrate to New Zealand. “Quality of life (or that of my family)” was indicated as “important” or “highly important” by 96% of respondents, 87% indicated more attractive working conditions and 72% said it was availability of career opportunities. Notably, 65% indicated a “desire to leave the UK NHS”, with one third of all respondents indicating that this was “highly important”. Only 38% agreed that “more attractive salary and incentives” motivated their move, with less than 10% saying this was highly important.

**Table 2:** **Respondents’ motivations to migrate to New Zealand (** ***n*** **= 632)**

**Item**	**Unimportant (%)**	**Not very important (%)**	**Important (%)**	**Highly important (%)**	**Important or highly important (%)**
Quality of life (or that of my family)	2	2	28	68	96
More attractive working conditions	5	8	37	51	87
Availability of career opportunities	7	22	53	18	72
Personal/family factors	9	20	33	38	71
Desire to leave the UK NHS	11	24	35	30	65
My training and development goals	10	33	44	14	57
My career progression goals	11	36	40	13	52
More attractive salary and incentives	16	46	33	5	38
Desire to return to New Zealand, a country I had previously worked/lived in	55	16	16	12	29
Capacity to work in both public and private practice	67	24	7	2	9

The final column is the sum of “Important” and “Highly important” responses. Note percentages may not total because of rounding.

Table 3 features results of POM analyses for three questions of interest. These highlight that older respondents (aged 41 years and above) were less inclined to agree than 20–30 year olds (the reference group) that quality of life was an important motivator. Registrars were also less likely than GPs to be seeking a better quality life but over twice as likely as GPs to be motivated by “training and development goals”. When it came to the desire to leave the NHS, younger doctors (20–30 years of age) were significantly more likely than older doctors (aged 51 and over) to agree that this was a motivating factor.

**Table 3:** **POMs describing associations between respondent demographic characteristics and factors important to motivating them to migrate to New Zealand (quality of life in New Zealand, their training and development goals and a desire to leave the UK NHS)**

	**Quality of life**			**Training and development goals**			**Desire to leave NHS**		
	**OR**	**95% CI**	***P***	**OR**	**95% CI**	***P***	**OR**	**95% CI**	***P***
Gender									
Female	Reference								
Male	1.07	0.73–1.58	0.7197	1.03	0.73–1.44	0.8857	1.27	0.91–1.76	0.1566
Age									
20–30	Reference								
31–40	0.78	0.41–1.45	0.4253	1.55	0.89–2.72	0.1253	0.60	0.35–1.01	0.0564
41–50	0.33	0.12–0.92	0.0350*	1.01	0.42–2.43	0.9885	0.61	0.25–1.48	0.2673
51–60	0.08	0.02–0.29	0.0001*	0.22	0.07–0.67	0.0082*	0.24	0.08–0.76	0.0149*
61+	0.05	0.01–0.21	0.0001*	0.15	0.04–0.55	0.0045*	0.22	0.06–0.83	0.0266*
Years practising									
5 or less years	Reference								
6–10 years	0.97	0.51–1.85	0.9354	0.79	0.45–1.39	0.4113	1.38	0.81–2.36	0.2378
11–15 years	0.84	0.36–1.99	0.6944	0.60	0.28–1.30	0.1966	1.16	0.56–2.39	0.6912
16–20 years	1.63	0.52–5.22	0.4082	0.26	0.10–0.70	0.0071*	0.80	0.30–2.12	0.6611
21 or more years	3.64	1.08–12.54	0.0384*	0.29	0.10–0.81	0.0177*	1.25	0.45–3.49	0.6683
Main job									
General practitioner	Reference								
Hospital specialist	0.67	0.37–1.22	0.1985	1.54	0.94–2.55	0.0894	1.44	0.88–2.36	0.1419
Registrar	0.40	0.21–0.75	0.0051*	2.24	1.30–3.86	0.0035*	1.21	0.72–2.05	0.4755
Other	0.53	0.26–1.10	0.0911	1.66	0.88–3.11	0.1151	0.64	0.35–1.18	0.1543

Gender, age (categorized), years practising (categorized) and main job were included as variables in each multivariate POM.

*P < 0.05.

Table 4 displays responses to items pertaining to respondents’ work and living environment in New Zealand, factors deemed important to workforce sustainability. Respondents were a relatively content group with over 90% satisfied with their workload, work colleagues and community life and with the New Zealand health system being “easy to work in”. Eighty per cent agreed that “The New Zealand health system is better to work in compared to the UK system”, with over 40% strongly agreeing with this statement. Regression results in Table 5 revealed males and older respondents (41 years and over) were less likely to agree with this statement, while hospital specialists and registrars were more likely to agree than GPs.

**Table 4:** **Respondents’ perceptions of the working and living environment in New Zealand (** ***n*** **= 632)**

**Item**	**Strongly disagree (%)**	**Disagree (%)**	**Agree (%)**	**Strongly agree (%)**	**Agree or strongly agree (%)**
I get along well with my work colleagues	0	1	43	55	98
I enjoy living in my local community	0	2	40	58	97
I have good clinical support	1	5	59	35	94
I feel that my work is valued by the local community	1	5	54	40	94
The New Zealand health system is easy to work in	1	7	63	29	92
My workload is reasonable	2	7	56	35	91
I have good facilities and equipment to work with	1	8	63	27	90
Integration into the New Zealand health system was straightforward	2	9	60	29	89
I received adequate orientation and support on arrival in New Zealand	3	13	62	22	84
The New Zealand health system is better to work in compared with the UK system	2	18	40	40	80
I have good administrative support	5	20	55	19	74

The final column is the sum of “Agree” and “Strongly agree” responses. Note percentages may not total because of rounding.

**Table 5:** **POM describing associations between respondent demographic characteristics and agreement with the statement “The New Zealand health system is better to work in compared to the UK system”**

	**OR**	**95% CI**	***P***
Gender			
Female	Reference		
Male	1.62	1.17–2.26	0.0041*
Age			
20–30	Reference		
31–40	0.58	0.33–1.02	0.0591
41–50	0.33	0.14–0.81	0.0155*
51–60	0.15	0.05–0.45	0.0007*
61+	0.15	0.04–0.56	0.0049*
Years practising			
5 or less years	Reference		
6–10 years	1.21	0.68–2.16	0.5211
11–15 years	0.79	0.38–1.67	0.5396
16–20 years	0.92	0.35–2.43	0.8589
21 or more years	1.13	0.41–3.12	0.8121
Main job			
General practitioner	Reference		
Hospital specialist	2.20	1.36–3.58	0.0014*
Registrar	2.51	1.50–4.24	0.0005*
Other	1.26	0.71–2.25	0.4306

Gender, age (categorized), years practising (categorized) and main job were included as variables in the multivariate POM.

*P < 0.05.

Twenty-nine per cent of respondents indicated that they were considering a move away from New Zealand. This subset (*n* = 181) were then asked to rate their level of agreement or disagreement with a series of considerations. Table 6 contains the findings. At 76%, the highest scoring factor was “desire to return to a country (e.g. UK) where I had previously lived/worked”. Next in order of importance, at 55% agreement, was availability of career opportunities elsewhere. Some 24% were motivated by “more attractive salary and incentives” elsewhere and 20% by a “better lifestyle elsewhere”; only 15% cited a “poor working environment” in New Zealand as being a consideration.

**Table 6:** **Importance placed on various reasons for moving away from New Zealand in respondents indicating that they were considering leaving New Zealand (** ***n*** **= 181)**

**Item**	**Unimportant (%)**	**Not very important (%)**	**Important (%)**	**Highly important (%)**	**Important or highly important (%)**
Desire to return to a country (for example, UK) where I had previously worked/lived	11	13	36	40	76
Availability of career opportunities elsewhere	25	20	39	16	55
Limited career opportunities in NZ	31	28	25	16	40
More attractive salary and incentives elsewhere	43	33	14	10	24
More attractive conditions of work elsewhere	43	35	16	5	21
Better lifestyle elsewhere	50	30	17	3	20
A poor working environment in NZ	60	25	12	4	15
Lack of administrative support staff in NZ	60	31	7	2	9
Lack of clinical support staff in NZ	60	32	8	0	8

The final column is the sum of “Important” and “Highly important” responses. Note percentages may not total because of rounding.

## Discussion

This survey study of IMGs born or trained in the UK and practising in New Zealand elicited several findings which both corroborate and build on the knowledge generated from other studies of doctor migration. Key findings were that respondents were primarily motivated by “pull” factors that also motivate IMGs moving from lower to higher income countries: quality of life, better working conditions and career opportunities [3,10]. A crucial finding of relevance to UK policy makers and NHS leaders was the high proportion of respondents (65%) who had indicated a desire to leave the NHS as a primary motivation for seeking work in NZ, which could be considered a critical “push” factor. This was a much higher proportion than found in a prior study of NHS exports to New Zealand where 16% of respondents cited “disillusion with the NHS” [20]. That said, the comparison of findings between these two studies needs to be treated with caution, given different survey designs. If the number of doctors seeking to escape the NHS has increased over time, one explanation could be the increasing financial and patient demand pressures [38]. Particularly troubling of the present study’s findings was the significantly higher odds of younger doctors being propelled from the NHS, as well as by seeking a better quality of life. This, of course, parallels with reports elsewhere of younger doctors wishing to build more balance into their work and personal lives [20,24]. If such findings are a concern for policy makers in the UK, then they imply that more attention needs to be paid to the alleged workforce stress in the NHS and to ensuring working arrangements which satisfy the changing aspirations of younger doctors [27]. Of concern to policy makers in New Zealand was the finding of a much higher likelihood of respondents who were more recent arrivals indicating a possible intent to move away.

The survey probed respondents’ experiences living and working in New Zealand. In theory, when these experiences are positive, the length of workplace tenure should increase [19,39,40]. The findings provided limited insight into why, as was noted in the “Background” section, UK doctors stay for such a short time in New Zealand. Indeed, the findings suggest that respondents were predominantly relatively satisfied with all elements of work and living conditions. This points to two possibilities: that doctors from the UK seek work in New Zealand either with initial plans for taking up a longer term residence as reported elsewhere [20], and end up enjoying their adopted home, but find the “pull” of home and family reduces their length of stay or that they only ever intend to stay for a short time. The survey findings tend to support the former possibility. If the pull of home is a primary motivator behind the high turnover of UK doctors in New Zealand, then there may be little that New Zealand policy makers can do to counter this. One question not able to be probed in this study was why a higher proportion of respondents (29%) were considering moving away from New Zealand, compared with an earlier study’s finding that 89% of respondents “definitely or probably intended to continue to practice” in New Zealand [20]. This would tend to indicate an increase in desire to depart New Zealand, along with increasing numbers of doctors seeking refuge in New Zealand from the NHS. Such questions point to the need for further research but also improved routine data collection as noted elsewhere [5]. This could include exit and entry surveys administered by registration bodies that would permit closer analysis of workforce migration patterns and contributing factors. Questions around country of origin and intended destination could also be further investigated. This study’s sample was doctors with UK origins. As found elsewhere in the case of migration away from Ireland, this does not necessarily mean this is where all of these doctors were originally from [5].

The UK has a long tradition of supplying doctors to New Zealand dating back to early colonial settlement and, in 1875, the founding of its first medical school [41]. In this context, the UK doctor going down under to New Zealand for a limited period of time is nothing new. There has been increasing recognition that New Zealand’s reliance on IMGs is not ideal [21,42]. The public hospital doctors union has campaigned vigorously for growing the local workforce [22,31]; the government, also, has acknowledged the need for additional investment [23]. Since 2007, training places in New Zealand’s two medical schools have almost doubled. However, New Zealand continues to produce fewer medical graduates than the OECD average. Its doctor-to-population ratio of 2.6:1000 population is also below the OECD 3.2:1000 average [4].

As New Zealand works to grow its medical workforce to keep pace with health care demands, it is likely, in the short term, to continue relying heavily on IMGs, especially in areas with crucial shortages such as rural general practice and psychiatry [23]. Doctors with these skills seeking time out in New Zealand have little to worry about. However, the situation could change quite rapidly for two reasons. First, the market in Australia, a traditional “life in the sun” for New Zealand doctors, is tightening up as it graduates doctors from 10 new medical schools established since 2000 [43]. Second, new schemes to keep New Zealand doctors at home after graduation – including bonding and reimbursement of university fees for graduates who agree to work in hard-to-staff areas – are starting to have an impact, along with the increased medical school output [23].

The limitations of this study need acknowledgement. These include the response rate of 47% and fact that we know little about non-respondents. However, as noted, a very high percentage of respondents completed the survey in full. Furthermore, it is widely recognized that achieving high response rates in studies of medical professionals and health workforce and service delivery issues is increasingly challenging [44-46]. In this context, 47% could be considered a good response. Second, there was a slight tendency in the respondent group towards older doctors in permanent posts, compared with the broader UK IMG workforce in New Zealand. This may have affected the findings. Third, this study included only two high-income counties, so there may be limits on the extent to which the findings are generalizable beyond the study setting. Fourth, the simple survey method, while providing valuable insights, did not allow for further examination of various issues. For example, the study focus on a single broad cohort did not permit for analysis of respondents by time of arrival in New Zealand and linking of this to time of training completion in the UK, thus allowing for time-related cohort analysis. The survey did not inquire into whether respondents had gone to the UK specifically to train in medicine then departed for New Zealand; following this, it also did not ask about the particular country respondents might be seeking to return to if planning to leave New Zealand. As with any fixed-response survey study, in-depth interviews and focus group discussions could reveal useful additional information [47].

## Conclusions

Keeping in mind the limitations outlined above, this study makes an important contribution to the literature on IMGs as it assists with understanding some of the reasons why doctors move between high-income countries. New Zealand has long relied on migrant doctors from the UK. However, the proportion of respondents in this study seeking to leave the NHS was higher than found elsewhere, possibly reflecting increasing workplace and funding pressures of recent years. Perhaps of greatest concern to policy makers was the higher odds of younger doctors being motivated by a desire to depart the NHS and then expressing an intention to move on from New Zealand. This aside, various changes “down under”, in New Zealand as well as Australia, mean their IMG markets may well be tightening up.
